# Molecular Response of Estuarine Fish to Hypoxia: A Comparative Study with Ruffe and Flounder from Field and Laboratory

**DOI:** 10.1371/journal.pone.0090778

**Published:** 2014-03-03

**Authors:** Jessica Tiedke, Ralf Thiel, Thorsten Burmester

**Affiliations:** Institute of Zoology and Zoological Museum, University of Hamburg, Hamburg, Germany; Universidade de Brasília, Brazil

## Abstract

On a global scale, the frequencies and magnitudes of hypoxic events in coastal and estuarine waters have increased dramatically over the past 20 years. Fish populations are suitable indicators for the assessment of the quality of aquatic ecosystems, as they are omnipresent and often comprise a variety of different lifestyles and adaption strategies. We have investigated on the molecular level the impact of hypoxia on two fish species typical of European estuaries. We monitored the expression of eleven putatively hypoxia-responsive genes by means of quantitative real-time RT-PCR in brains, gills and hearts of the ruffe (*Gymnocephalus cernua*) and the flounder *(Platichthys flesus*). We first investigated the effect of naturally occurring hypoxia in the Elbe estuary. In a second approach, expression changes in the response to hypoxia were monitored under controlled laboratory conditions. The genes that showed the strongest effect were two respiratory proteins, myoglobin and neuroglobin, as well as the apoptosis enzyme caspase 3. As previously observed in other fish, myoglobin, which was considered to be muscle-specific, was found in brain and gills as well. Comparison of field and laboratory studies showed that – with the exception of the heart of flounder – that mRNA levels of the selected genes were about the same, suggesting that laboratory conditions reflect natural conditions. Likewise, trends of gene expression changes under hypoxia were the same, although hypoxia response was more pronounced in the Elbe estuary. In general, the flounder displayed a stronger response to hypoxia than the ruffe, suggesting that the flounder is more susceptible to hypoxia. The most pronounced differences were found among tissues within a species, demonstrating that hypoxia response is largely tissue-specific. In summary, our data suggest that laboratory experiments essentially mimic field data, but additional environmental factors enhance hypoxia response in nature.

## Introduction

Environmental hypoxia is an increasing problem worldwide with severe consequences for aquatic ecosystems. Oxygen supply in water bodies is generally mediated by the diffusion of atmospheric oxygen and by the production of the photoautotrophic plants algae and phytoplankton [1], [2]. The concentration of dissolved oxygen (DO) depends on temperature, salinity and pressure. In shallow coastal areas and estuaries, environmental hypoxia has been increasing severely over the past decades [3]. These habitats are also characterised by various additional anthropomorphic stressors, such as hydro-morphological changes and excessive nutrient inputs as well as natural variations in turbidity and temperature, which might have synergistic impacts [4], [5].

Typically, environmental hypoxia is defined when the DO is below 2 mg O_2_/l. Under these conditions, the first effects appear in most aquatic organisms [3], [6]. Hypoxia mainly occurs during the summer months when the solubility of oxygen in water decreases due to the rising temperature. This effect is further enhanced by the stratification of the water column by formation of thermo- and haloclines [7]. In addition, eutrophication, which is mainly caused by the input of nitrogen and other nutrients through anthropogenic activities, may further reduce the available level of DO [8]. In combination with an insufficient vertical mixing rate through stratification, this leads to bottom-water hypoxia, which is common in estuaries [6], [9], [10], [11].

**Figure 1:**
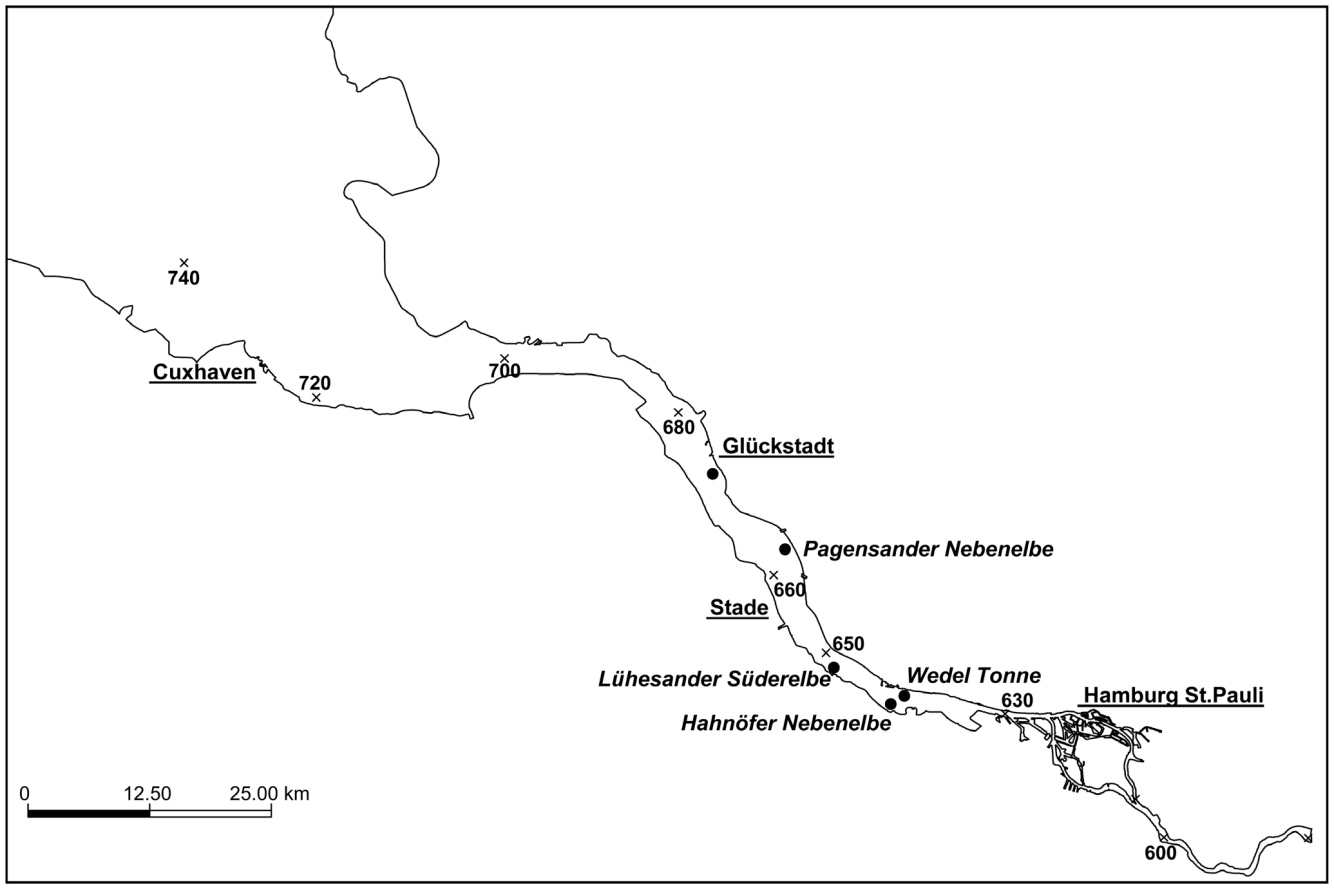
Map of the 140 km long Elbe estuary between Hamburg and Cuxhaven. Underlined names are cities along the estuary used as benchmarks. Names in italics identify the five sampling sites, which are additionally marked by black dots. Modified from Eick [99].

The Elbe is one of the longest European rivers (1,094 km) with a catchment area of ∼144,000 km^2^ that is dominated by agricultural land use [12]. The 140-km-long estuary is an important waterway and includes the harbour of the metropolitan region of Hamburg (Fig.1). These particular and permanently changing physical and chemical characteristics make the estuaries one of the most challenging environments for animal life [13]. Nevertheless, 78 fish species occur in the Elbe estuary, which is thus one of the most species-rich European estuaries [14]. In the last decades, the decreasing DO levels caused by the increased environmental temperature have additionally gained in severity due to a reduction of the surface/volume ratio by the deepening of the Elbe estuary for commercial shipping [14]. The oxygen budget of the Elbe estuary temporarily improved by reprocessing facilities and reduction of industrial sewage discharges after the German reunification. However, in recent years dredging of the river bed has lead to the recurrence of seasonal oxygen shortage in the Elbe estuary [15], [16].

The ectothermic and aerobic lifestyle of fish makes them in principle very susceptible to rising temperatures and decreased oxygen availability [17], [18]. This is also reflected by a large number of hypoxia-tolerant fish species that have emerged during evolution. On the behavioural level, fish may decrease their energy demands e.g. by reducing the swimming activity or avoidance of hypoxic areas by vertical or horizontal migration [19], [20]. On the morphological and physiological levels, some species have evolved specific adaptations that help them cope with hypoxic periods. These adaptations include, for example, the apoptosis-based expansion of the gill surface, the counter-current oxygen exchange and the increased oxygen affinity of fish haemoglobin, resulting in oxygen extraction efficiencies of 50–90% [21], [22], [23], [24]. On the molecular level, various fish species show the typical cellular responses which depend on the activation of the hypoxia-inducible transcription factor (Hif-1) or are part of the conserved cellular stress response [25].

The dramatically increased frequencies and magnitudes of hypoxic events in the past ∼20 years, are suspected to have major impact on the biodiversity of fish populations, leading to growing interest in better understanding the particular impact on fish physiology [9]. Here, we have investigated the response to seasonal hypoxia in the tidal Elbe of two typical estuarine fish species: the ruffe (*Gymnocephalus cernua*), which is an omnipresent species in temperate regions of Europe and northern Asia, and the European flounder (*Platichthys flesus*), which additionally is of commercial interest. Flounder and ruffe have divergent life histories and therefore may have assumed different adaptation to hypoxic periods. The flounder is a demersal fish with a mainly sedentary lifestyle, which lives in shallow coastal waters and estuaries [26], [27]. The ruffe is a more active predator, which inhabits freshwaters as well as brackish waters of rivers, lakes, and tidal estuaries [27], [28]. In general, it is assumed that sustained swimmers are more sensitive to oxygen deprivation than bentho/pelagic species that more regularly encounter low oxygen in their environment [29].

In this study, we characterized the species and tissue-specific hypoxia response by measuring mRNA levels of selected stress-response genes, such as members of the anaerobic energy metabolism, oxidative stress defence and oxygen supply. Effects of hypoxia were monitored in three tissues (gills, brain and heart). To evaluate the impact from the more complex environment of the Elbe estuary, we additionally conducted controlled hypoxia experiments in the laboratory.

## Materials and Methods

### Ethics Statement

Eurasian ruffe (*G. cernua*) and flounder (*P. flesus*) were collected with a commercial fishing vessel. No permit was required for commercial fishing. Both species are neither endangered nor protected. The experimental procedures for handling and killing of fish specimens were approved by the Hamburg authorities (Behörde für Soziales, Familie, Gesundheit und Verbraucherschutz, License No. 04/10).

### Field sampling

Adult ruffe (*G. cernua*) and flounder (*P. flesus*) were collected from five different sites in the Elbe estuary in Germany (Glückstadt, Pagensander Nebenelbe, Lühesander Süderelbe, Hahnöfer Nebenelbe, and Wedel Tonne; Fig. 1) during August and October of 2010 with a stow net cutter with a mesh size of 8 mm in the cod end. The DO and temperature data were taken at the beginning and end of each stow net haul with a multimode probe U 50 (Horiba, Japan). For each haul both values were averaged for further studies. In August, DO values correspond to mild hypoxia in Glückstadt (6.5 mg/l) and moderate hypoxia between Pagensander Nebenelbe (5.0 mg/l) and Wedel (4.2 mg/l) with an average water temperature of 23°C (Table 1). No sampling site showed severe hypoxic conditions (<2 mg/l). In October DO values in Glückstadt remained nearly saturated with 8.8 mg/l with an average water temperature of 14.7°C. After fishing adult flounders and ruffes were immediately collected from the fish haul without unnecessary suffering of the animals and divided into two groups. The group of specimen for laboratory experiments was maintained in the livewell, which was consistently provided with fresh aerated water from the surrounding, to ensure species-appropriate transport conditions. The field study group was instantly anesthetized in ice-cold water to prevent discomfort and pain right before killing by cutting the spinal cord and the aorta dorsalis. Dissected gill, brain and heart (atrium, ventricle) tissues were stored in RNAlater. The other fish specimens were used for laboratory experiments.

**Table 1:** Comparison of oxygen concentrations in the Elbe estuary and in laboratory experiments.

Condition	Elbe estuary				Laboratory	
	Sampling site (stream km)	Time	O_2_ (mg/l)	T (°C)	O_2_ (mg/l)	T (°C)
**mild hypoxia**	Glücksstadt (674)	August	6.5	23	5.2	12
**moderate hypoxia**	Pagensand NE (662)	August	5.0	23	3.6	11
	Wedel (638)	August	4.2	23.6		
**severe hypoxia**	––	––	––	––	1.5	11
**normoxia**	Glücksstadt (674)	October	13	4	11	11

### Laboratory hypoxia treatments and tissue sample preparation

For controlled hypoxia experiments in the laboratory, ruffe and flounder were acclimated for at least four weeks in a 750-litre aquarium with freshwater at 11°C and with a day/night light cycle of 11.5/12.5 h. During acclimation, individuals were fed daily with the common earthworm (*Lumbricus terrestris*). Hypoxia treatments were carried out in a 100-litre tank at 11°C for 48 h using four adult individuals for each hypoxic condition: severe hypoxia (1.5±0.1 mg/l DO), moderate hypoxia (3.5±0.1 mg/l DO), mild hypoxia (5.2±0.1 mg/l DO) and normoxia (11.0±0.1 mg/l DO). For the calculation of the oxygen concentrations used for laboratory hypoxia experiments we used the average oxygen content measured during different seasons in the Elbe estuary. To ensure comparability of field sampling and laboratory experiments in spite of temperature differences the oxygen concentrations were calculated by percentage based on normoxia corresponding to 100% of dissolved oxygen (personal communication D. Eick and the past two years; BMU, 2010) (Table 1). Hypoxic conditions were reached in the aquaria within 1 h. Oxygen concentrations were measured using the Oxi 340i sensor (WTW, Weilheim, Germany). Hypoxic conditions were adjusted using the Roxy-1 controller (Sable Systems, Las Vegas, Nevada, USA), which was connected to a source of gaseous nitrogen. During hypoxia treatments fish were not given any food. After hypoxic treatments the animals were anesthetized and killed as mentioned above. Gills, brains and hearts were excised and immediately frozen in liquid nitrogen.

### RNA isolation and reverse transcription-polymerase chain reaction (RT-PCR)

For preparation of RNA, fresh tissue was ground to a fine powder in liquid nitrogen using a mortar and pestle. Total RNA was isolated with peqGOLD TRIfast™ (Peqlab, Erlangen, Germany) in combination with the RNeasy® Mini Kit and including an on-column digestion with RNase-free DNase (Qiagen, Hilden, Germany). Total RNA was quantified spectrometrically with the Nanodrop ND 100 UV-Vis spectrometer (Thermo Scientific, Bonn, Germany). RNA integrity was checked by using formaldehyde agarose gel electrophoresis. cDNA was synthesised with 1.5 µg total RNA using oligo-(dT)_18_ nucleotides and SuperScriptTM III Reverse Transcriptase (Invitrogen, Karlsruhe, Germany) according to the manufacturer's protocol.

### Sequence identification of genes of interest and specific primer design

Genes of interest (GOI) were amplified using degenerate oligonucleotides, which had been designed according to aligned nucleotide sequences from various members of Perciformes and Pleuronectiformes (Table S1). Amplification was performed via gradient PCR using 40 amplification cycles (95°C 1 min, 48°C–55°C, 45 sec, 72°C 1 min, 72°C 10 min). PCR fragments were cloned into the pGEM-T easy cloning vector (Promega, Mannheim, Germany) and sequenced by a commercial service (GATC, Konstanz, Germany). The sequences were then used for the design of specific primers. After amplification, the cDNA fragments of the GOI (80 to 200 bp) (Table S2) were cloned and sequenced. The recombinant plasmids with the GOI were used for the standard curves in qRT-PCR.

### Quantitative real-time RT-PCR

Quantitative real-time RT-PCR (qRT-PCR) experiments were carried out with an ABI 7300 Real-Time PCR system. The ABI Power SYBR Green Master Mix (Applied Biosystems, Darmstadt, Germany) was applied with a two-step protocol. We used cDNA equivalent to 37.5 ng total RNA for each reaction. Eleven putatively stress-regulated genes (hypoxia inducible factor 1 α, Hif1α; heat shock protein 70, Hsp70; heat shock protein 27, Hsp27; myoglobin, Mb; neuroglobin, Ngb; globin X, GbX; lactate dehydrogenase, Ldh; phosphoglycerate kinase, Pgk; superoxide dismutase 2, Sod2; caspase 3, Casp3; glutathione peroxidase, GPx) were investigated. The reference genes, β-Actin (β-Act), ribosomal protein large P0-like protein (Rplp0) and elongation factor 1α (Ef1α), were employed as putatively non-regulated controls. qRT-PCR was carried out with a standard cycling protocol using 40 amplification cycles (95°C 10 min, 95°C 15 sec, 60°C 15 sec; 72°C 30 sec). Success and specificity of amplification was evaluated using dissociation curves with a temperature range from 60°C to 95°C. All reactions were performed in triplicates. For standard curves, duplicates were run using tenfold serial dilutions (10^6^–10^2^) of recombinant plasmids representing GOI.

### Evaluation of qPCR data

We used a clustered structure to analyse gene expression changes: Three biological replicates of each species and tissue were employed. The samples were split into two RT reactions and we finally used three replicates in qRT-PCR (modified according to [30]. First evaluation of qRT-PCR data was performed with the ABI 7300 Sequence Detection software V.1.3.1 (Applied Biosystems). Reaction efficiency was determined by the slope of the standard curve for each gene. For evaluation of relative expression levels with the -δδCt method [31], mean values of all triplicates were used. Calculations were carried out with Microsoft Office Excel XP. The data were further evaluated with IBM SPSS Statistics 20. The distribution of variables and the equality of variances were determined using the Kolmogorov-Smirnoff test and Levene's test, respectively. In case of homogenic variances, one-way ANOVA followed by Dunnett's t-test was applied, whereas in the case of heterogeneity a one-way ANOVA using the Games-Howell correction for post-hoc analysis was performed (Table S3A–C). To further evaluate the overall effect of field versus laboratory experiments and species specificity on gene expression, we performed a multivariate analysis of variance (MANOVA). We used the Pillai trace statistic, which is robust even with small sample sizes [32]. The MANOVA contained the effects of the setups (field versus laboratory) for ruffe and the effect of species specificity within a tissue and referred to one hypoxic condition (Table S4).

## Results

We investigated the molecular response to hypoxia in the ruffe and in the flounder by qRT-PCR employing a set of genes that represent a broad range of cellular functions. First, we obtained the partial coding sequences of the GOI (Table S2), with the exception of Hsp27 from the flounder, for which no specific cDNA could be amplified. Evaluation of the mRNA levels of the three putative reference genes (see above) revealed that RPLP0 expression was not affected by the hypoxic conditions in both species. Thus, RPLP0 was used for the normalisation of the qRT-PCR data.

For each field site and experimental laboratory condition, groups of three fishes were used. Each tissue sample was used for two independent extractions. Thus each expression data set corresponds to three biological with two technical replicates each (n = 3×2).

### Gene expression in ruffe and flounder under normoxia

**Figure 2:**
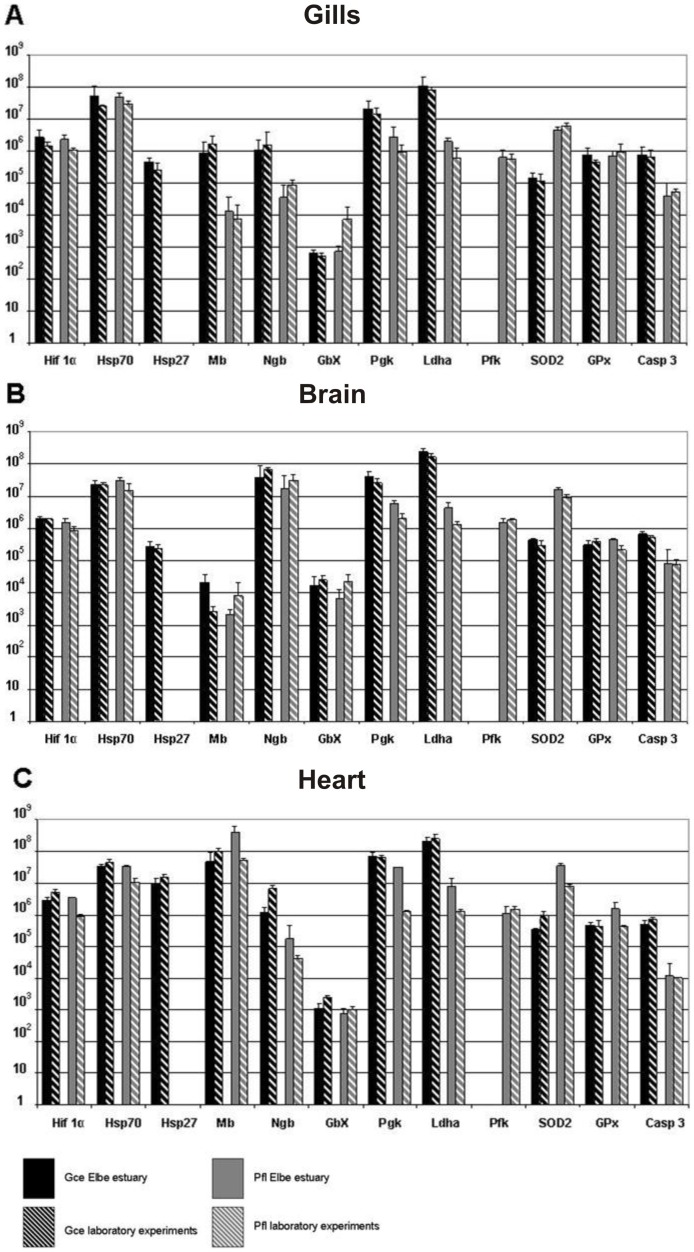
Interspecies and experimental site comparison of total mRNA copy numbers of putatively hypoxia responsive genes in ruffe and flounder under normoxic condition analysed in (A) gills, (B) brain and (C) heart. All expression levels are referred to 1 µg mRNA. qRT-PCR results from the Elbe estuary are represented in black columns for ruffe and grey columns for flounder. Dashed columns indicate qRT-PCR results from laboratory experiments with black background for ruffe and grey background for flounder, respectively. Note that the y-axis has a logarithmic scale. Bars indicate standard deviation (SD). Asterisks indicate significance at p <0.05. N = 3 individuals for results from Elbe estuary for each data point. N = 3 (analysed with a nested experimental design) for results from laboratory for each data point.

Under normoxic conditions (defined in our experimental setup as 13 mg/l DO at 4°C in the Elbe estuary and 11.0±0.1 mg/l DO at 11°C in the laboratory), there were essentially no significant differences in terms of total mRNA copy numbers in both species (Fig. 2).

As expected, we found the highest level of Mb, which is the typical respiratory protein of the striated muscles [33] in the hearts (∼10^8^ copies per µg total RNA). Likewise, mRNA levels of the neuronal respiratory protein Ngb [34], [35] were highest in the brains (∼10^8^ copies per µg total RNA). Nevertheless, minor amounts of Mb mRNA were also observed in gills and brains as well as Ngb mRNA in gills of both species.

Interspecies comparison revealed significant differences in the total copy numbers of some genes. In particular, transcript levels of Mb and Ngb were markedly different between species and tissues. For example, while we found approximately the same amounts of Mb and Ngb in the brains of the flounder and the ruffe (∼10^8^ copies/µg), in the gills of the ruffe the levels of these two genes exceeded those measured in the flounder by at least two orders of magnitude (Fig. 2A and 2B). Ngb copy numbers were twofold higher in the hearts of the ruffe than in the flounder (Fig. 2C). Likewise, the levels of Ldha, Pgk and Casp3 were on average about tenfold higher in the tissues of the ruffe whereas the copy numbers of Sod2 were higher in tissues of the flounder (about 40-fold on average) (Fig.2). Absolute expression levels of Hsp27 were about 15-fold higher in the heart of the ruffe compared to the gills and brains (Fig. 2).

A comparison of mRNA copy numbers in specimens from laboratory experiments and field studies showed that there are surprisingly little differences. Only in the flounder were significantly higher mRNA levels of Mb, Ldha, Pkg and Sod2 observed in the hearts of individuals taken from the Elbe estuary. By contrast, we found significantly higher Mb mRNA levels in the brains of the ruffe kept under laboratory conditions, although the total amount of Mb mRNA was low in this tissue.

### Gene expression changes under hypoxic conditions

**Figure 3:**
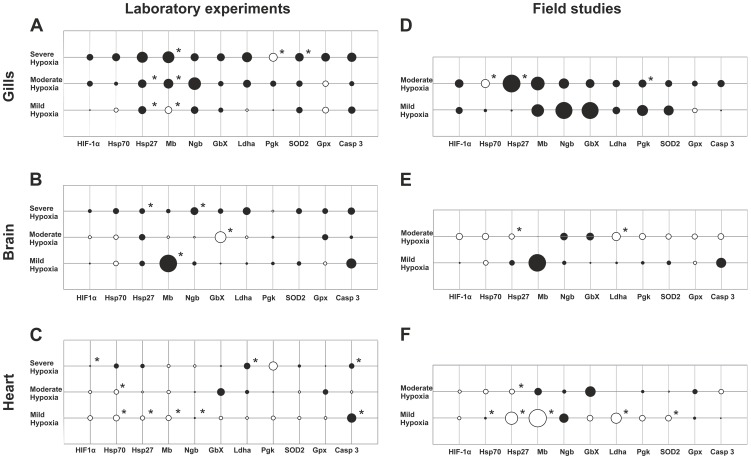
Bubble charts of relative expression patterns of putatively hypoxia responsive genes in ruffe under different hypoxic conditions in laboratory experiments (A, B and C) and in the Elbe estuary (C, D and E). Evaluation of qRT-PCR results was performed with ΔΔ CT method by use of RPLP0 as a reference gene. All results of relative expression levels are represented in log base 2. The diameter of bubbles indicates the magnitude of gene expression. Black bubbles represent expression levels >0, pen bubbles represent expression levels <0, and crossing lines without a bubble signify no change in the expression level. Asterisks indicate significance at p<0.1. We analysed the expression pattern in three tissues of ruffe: (A) and (D) gills, (B) and (E) brain and (C) and (F) heart. For more details see Table S2.

**Figure 4:**
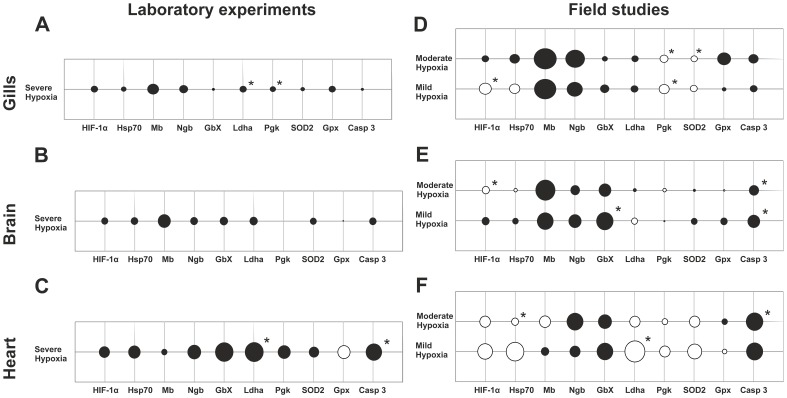
Bubble charts of relative expression patterns of putatively hypoxia responsive genes in flounder under severe hypoxic conditions in laboratory experiments (A, B and C) and different hypoxic conditions in the Elbe estuary (C, D and E). Evaluation of qRT-PCR results was performed with ΔΔ CT method by use of RPLP0 as a reference gene. All results of relative expression levels are represented in log base 2. The diameter of bubbles indicates the magnitude of gene expression. Black bubbles represent expression levels >0, pen bubbles represent expression levels <0, and crossing lines without a bubble signify no change in the expression level. Asterisks indicate significance at p<0.1. We analysed the expression pattern in three tissues of flounder: (A) and (D) gills, (B) and (E) brain and (C) and (F) heart. For more details see Table S2.

Interspecies comparisons indicated that the changes of gene expression levels under hypoxic conditions were more pronounced in the flounder in tissues. Here, 51% of all collected data points (ten genes/three hypoxic stages/three tissues) showed ≥ twofold regulation, of which 12% were statistically significant. In the ruffe, only 32% of data points (11 genes and five hypoxic stages/three tissues) with ≥ twofold regulation were found, of which 22% were significant (Fig. 3). A comparison of the responses between tissues showed the same tendencies in gene expression changes in gills of both species (Fig. 3 and 4 A and D). In the ruffe, the gills were the most stress-responsive tissue, with 49% of data points showing a ≥ twofold regulation (43% in the flounder) (Fig. 3 and 4 A and D). By contrast, in the flounder we found the strongest changes in the hypoxic brain with 56% of data points having a ≥ twofold regulation (20% in the ruffe) (Fig. 3 and 4 B and E). While we found as few as 20% of the data points regulated in the hearts of the ruffe, about 53% were found in the flounder (3 and 4 C and F). Notably, comparison of the data from both setups suggests that the overall response to hypoxia is more pronounced in samples taken from the Elbe estuary (Fig. 3 and 4). We used MANOVA for the evaluation of an overall effect of both setups and species specificity on the expression of all genes. We found no significant effect of the setups on gene expression. The MANOVA approach on the species specificity showed no significant effect in hearts, but the statistical tests in the gills and brains, however showed a significant effect in terms of moderate and severe hypoxia (Table S4).

### Analysis of putative stress-response genes in ruffe and flounder

Enhanced mRNA levels of the transcription factor Hif1α were observed in the gills of the ruffe from both setups (Fig. 3 A and D), and in brains, gills and hearts of the flounder under severe hypoxia in the laboratory (Fig. 4. A, B and C). Field study results showed that mRNA levels were slightly elevated in the gills of the ruffe, but rather unaffected in the flounder (Fig. 3 and 4 A and D). In the hearts and in the brains of both species from the field studies no clear trend was observed (Fig. 3 and 4 C and F).

Expression changes of the molecular chaperone Hsp70 were stronger in the flounder than in the ruffe. The flounder gills showed the strongest response (up to threefold upregulation) (Fig. 4 A). In the ruffe, Hsp70 expression was essentially unaffected by mild hypoxia (Fig. 3). However, under laboratory conditions severe hypoxia causes enhanced Hsp70 levels in all tissues of both fish species. As mentioned, Hsp27 sequences could only been obtained from the ruffe. In the gills of this fish, Hsp27 levels were found to be significantly enhanced at all hypoxic conditions in the field and in the laboratory (up to ∼50-fold) (Fig. 3A–E). In the other tissues, the effects were minor, with the notable exception of significantly lower Hsp27 levels in the hearts (Fig. 3C).

**Figure 5:**
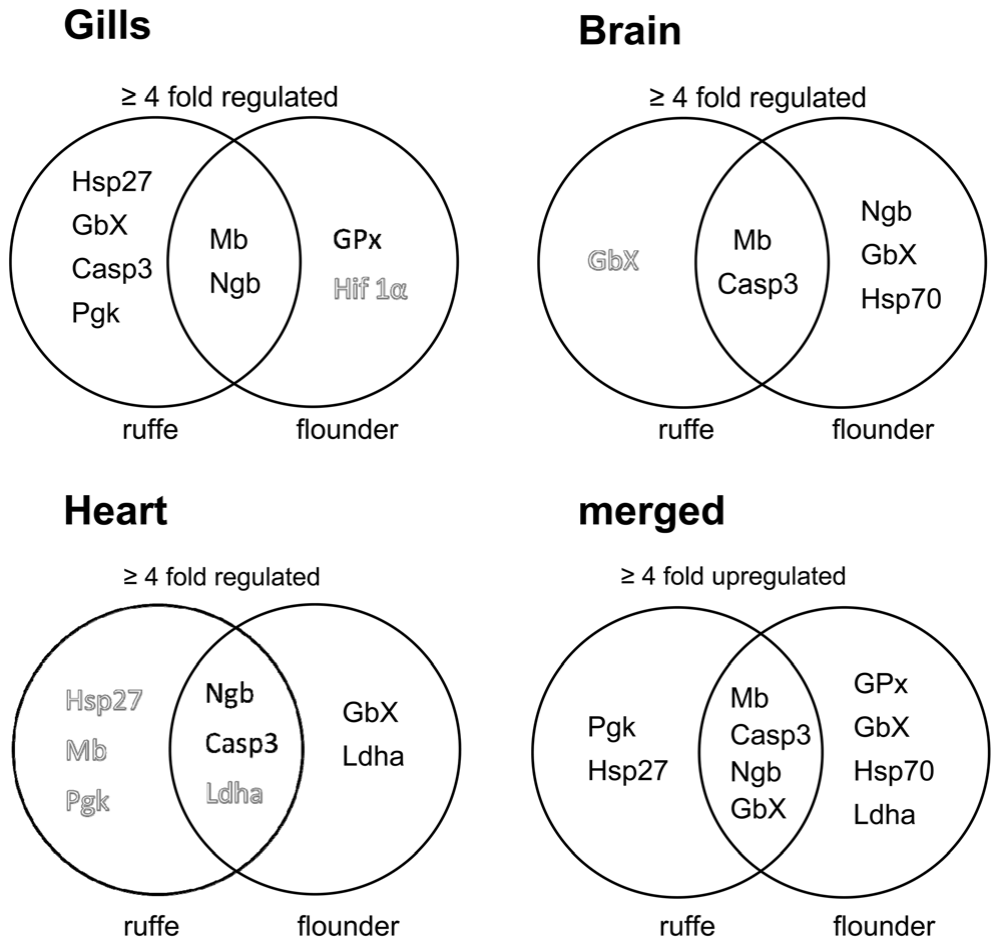
Venn diagram of expression changes in different tissues of ruffe and flounder. Black letters indicate genes that are upregulated and white letters indicate genes that are downregulated. Merged Venn diagram shows an overview of genes regulated more than fourfold in an overall comparison of the collected data.

We included three globins in our studies with putatively divergent functions: Mb, Ngb and GbX [33], [34], [36]. Hypoxia causes increased levels of the three globins in the gills of both species (all hypoxic conditions; both experimental sites) (Fig.3 and 4; Fig. 5). In this tissue Mb displayed the strongest response to hypoxia, with significantly higher levels in the ruffe (up to tenfold) and up to 160-fold in the flounder in the samples from the Elbe (Fig. 3 and 4 A and D). Interestingly, we also found an up to 200-fold increased Mb level in the brains of the flounder (moderate hypoxia; field studies) and laboratory experiments (severe hypoxia; up to ninefold) (Fig. 3 and 4 B and E; Fig. 5). However, in the hearts, which have high endogenous Mb levels, there is no upregulation of Mb mRNA. Rather, we found the Mb levels unchanged, or even more significantly, up to 100-fold lower (ruffe: mild hypoxia; field experiments) (Fig. 2C). Hypoxia causes an increase of Ngb mRNA levels in all tissues of both species. The strongest response was observed in the gills of both species from the Elbe estuary (up to 41-fold upregulation) (Fig. 3 and 4 A and D; Fig. 5). For GbX, we observed divergent changes of expression levels in the two fish species. In the brains, we found significantly elevated levels under hypoxic conditions in the flounder (up to 55-fold; field studies), but no changes in the ruffe. By contrast, similar tendencies were observed in the hearts of both species; here, transcript levels were elevated up to 4.5-fold in the ruffe (field studies) and up to sixfold in the flounder (laboratory experiments) (Fig. 3 and 4 C and F).

Likewise, the expression changes of the energy metabolism enzymes Pgk and Ldha showed divergent patterns (Fig. 3 and 4). Under severe hypoxic conditions, Ldha mRNA levels were elevated in all tissues of both species. In the ruffe we found enhanced Ldha levels in the gills (all hypoxic conditions; both experimental setups), while the levels significantly declined in the brains (mild and moderate hypoxia; field sampling) (Fig. 3A and B). In the flounder Ldha mRNA levels were slightly elevated in brains and showed significant upregulation in the hearts (severe hypoxia; laboratory experiments) (Fig. 4 D–F; Fig. 5). We found that Pgk was only affected in the gills of both species. Here, we observed that expression levels were significantly decreased in the flounder (about twofold), but significantly increased in the ruffe (about twofold) (Fig. 3 and 4 B and E).

The expression levels of antioxidant enzymes (Sod2; GPx) were mostly affected in the gills of both species. Here, we observed a 2.5-fold upregulation of Sod2 and GPx (severe hypoxia; laboratory experiments) and an up to sixfold upregulation of GPx in flounder (moderate hypoxia; field studies) (Fig. 3 and 4 A and D). The apoptosis enzyme Casp3 showed significant upregulation in the brains and hearts of the flounder (all hypoxic conditions; both experimental setups) (Fig. 4 D–F). The strongest expression changes were observed in the brains (up to eightfold) and in the hearts (up to sevenfold) of the flounder (Fig 4 E and F). In the ruffe we found significantly up to fivefold increased levels of Casp3 in the hearts (mild and severe hypoxia; laboratory experiments) and similar tendencies in the brains (Figs. 3–5).

## Discussion

Oxygen availability is one of the most important environmental factors that influence fish biodiversity, particularly in coastal waters and estuaries. Hence, understanding the impact of oxygen limitations provides insights into the consequences for fish populations and whole ecosystems. We investigated the molecular responses in the ruffe (Perciformes) and the European flounder (Pleuronectiformes). These two bony fishes belong to different orders of Percomorpha that diverged about 100 million years ago in the Cretaceous period [37]. Thus, our approach allows the comparison of species with divergent life histories and which therefore have adapted independently to the estuarine environment.

In this study we focussed on changes in mRNA levels of putatively stress genes in response to hypoxic conditions. We must emphasize that there may be post-transcriptional and translational processes additionally regulating protein abundances and activities, and that therefore the observed changes in mRNA levels do not necessarily match the changes in protein levels or enzyme activities.

### Oxygen sensing and metabolic changes in response to hypoxia

In fish the perception of the oxygen concentration in the environment is mainly accomplished by specialized cells in the gills [38]. On the molecular level, O_2_ sensing is mainly facilitated by the transcription factor Hif1 [39], [40]. In mammals Hif-target genes are mainly involved in anaerobic energy metabolism, erythropoiesis and angiogenesis [41], [42]. Analogous Hif-mediated mechanisms most likely also exist in fish [25], [43], [44], [45]. Although Hif-mediated O_2_-sensing mainly occurs on a posttranslational level, additional transcriptional regulation has already been shown in several fish species [46], [47], [48]. In this study Hif1α-levels were found upregulated in response to hypoxia in the gills of both the ruffe and the flounder. As the main tissue for oxygen sensing and because of their direct exchange with hypoxic waters, the gills are actually expected to experience the most pronounced changes.

Hypoxia poses a major problem the animal's energy budget because the most efficient ATP supply is accomplished by aerobic pathways. The metabolic responses to hypoxia may be distinguished by the means of meeting energy demands [49]. One strategy relies on the reduction of the energy demand by reducing protein synthesis, protein degradation and ion-pumping. This strategy is typical for anoxia-tolerant fish such as the goldfish [50], [51]. On the molecular level genes required for the oxygen-dependent energy production (e.g., the TCA cycle or the respiratory chain) and protein translation were repressed [52], [53]. Another strategy is the compensation of decreasing aerobic energy production by an increase of anaerobic ATP generation [44]. Therefore, genes coding for enzymes of the glycolytic pathway and fermentation were found more strongly expressed under hypoxic conditions in several fish species [43], [48], [53]. Our data provide evidence that both species, ruffe and flounder, meet their energy demands by the latter strategy as they show increased expression levels of the two glycolytic enzymes Ldha and Pgk, particularly under severe hypoxic conditions.

The energy production at the mitochondrial respiratory chain is known to be the major source of reactive oxygen species (ROS) [54]. Under low oxygen conditions electron transport slows down, which increases the reduction state of electron transporters and in turn favours superoxide production by increasing the electrical potential for single electron reduction of oxygen [55]. An increased formation of ROS may cause the damage of macromolecules [56]. The cellular homeostasis is protected by a defence system that consists of antioxidant compounds as well as antioxidant enzymes. In our assays, the levels of the antioxidant enzymes Sod2 and GPx were essentially unaffected by hypoxia. Welker et al. [57] emphasized that under hypoxic conditions there are different strategies to cope with ROS. While some species increase the activity and mRNA levels of antioxidant enzymes, others show little response to hypoxia. This might be explained by constitutively high levels of defence enzymes or insufficient hypoxia-tolerance [57]. Notably, only in the gills of ruffe Sod2 mRNA levels were found to be significantly increased, whereas we found minor upregulation of GPx in the gills of flounder from the Elbe estuary.

It should also be mentioned that thermal stress itself induces increased production of ROS in mitochondria because of a lower ADP to oxygen ratio, particularly in ectotherms [58], [59]. Some studies on temperature effects show increasing activities of Sod but not GPx in fish [60], [61], [62]. Notably, Kammer et al. [61] found Sod activation prior to an upregulation of transcript levels and suggested that Sod activity is likely depends on posttranslational modification, which we do not see with our studies. Therefore, the mRNA levels of the antioxidant enzymes are at best a clue and we cannot actually exclude cellular response to oxidative stress in the hypoxic ruffe and flounder.

### The specific role of globins in the hypoxia-response of ruffe and flounder

Globins are small metallo-proteins that reversibly bind O_2_ and thus are at the interface between the organism and its environment. Therefore, globins are a prime tool for studying the response of fish to hypoxia [44], [63], [64], [65], [66], [67], [68].

In addition to haemoglobin, which transports in the blood, there are four other globin types in the teleost fishes: Mb, Ngb, Cygb and GbX [69], [70], [71]. In most vertebrates, Mb appears to be restricted to the heart and skeletal muscles [33]. However, recent studies have suggested that Mb is also expressed in non-muscle tissue of various Cypriniformes [64], [67], [72] and medaka [68]. While by far the highest levels of Mb were observed in the hearts of the ruffe and the flounder (10^8^ copies/µg total RNA), notable amounts of Mb mRNA were also found in the gills and in the brains of these species. Thus, there may be a specific function of Mb in these tissues, which may relate to the O_2_ supply, the detoxification of ROS or the regulation of blood flow by scavenging NO [68], [72]. Notably, there is little response of Mb to the hypoxic conditions we applied here in the hearts (Fig. 3 and 4). This may be explained at least in part by the already high levels of endogenous Mb mRNA in tissue (Fig. 2). Interestingly, Mb mRNA was found upregulated in non-muscle tissues of both species, with statistical significance in the gills of the ruffe. Whether this observation relates to a specific role of Mb in these tissues remains to be established.

Although its exact function is still uncertain, Ngb is considered to be involved in the oxidative metabolism of neurons [34], [35]. It was therefore expected that Ngb levels were highest in the brains (∼10^8^ copies mRNA/µg total RNA; Fig. 2). These values are much higher than those found in the medaka [68], in which Ngb most likely only plays a minor role in hypoxia adaptation. Notably, the levels of Ngb mRNA were among the highest found in this study and within the range of Mb in the hearts. This finding rather contradicts notions that Ngb is a lowly expressed gene [73], [74], but confirms previous findings in zebrafish [75]. The relatively high levels of Ngb in gills and hearts may be explained by its expression in the peripheral nervous system [76]. However, in zebrafish Ngb has also been detected in the mitochondria-rich chloride cells of the gills, which presumably consume a large amount of energy [77]. The pattern of hypoxia response of Ngb in vertebrates is not clear [63]. In the flounder enhanced Ngb mRNA levels were found under hypoxia in all analysed tissues. In the ruffe, mRNA levels were elevated in the gills and significantly upregulated in the brains (Fig. 3). This corresponds to the findings in zebrafish [66], while no hypoxia response of Ngb was found in goldfish [67] and medaka [68]. Thus, the hypoxia response of Ngb in fish and its role in hypoxia may be species-specific.

GbX is a globin that is bound to the cell membrane [36] and has a widespread occurrence in the animal kingdom [78]. Most likely, GbX has no respiratory function, but may be either involved in the protection of membrane lipids or in some undefined signalling pathways [36]. In comparison to Mb and Ngb, GbX is rather lowly expressed (Fig. 2). The highest GbX levels were found in the brains, which is in agreement with its predominantly neuronal expression [36]. Hypoxia response of GbX has previously been investigated in the zebrafish, demonstrating its downregulation [66]. While in the ruffe no specific trend was observed, in the flounder hypoxia causes an increase of GbX mRNA in brains and hearts (Fig. 3). As with Ngb, hypoxia regulation of GbX may be species-specific, and the changes in flounder part of the enhanced hypoxia response found in this species.

### Species-specific responses of ruffe and flounder to hypoxia

Lifestyle is one of the main factors determining the hypoxia-tolerance of fish species. As stated above it is assumed that bentho/pelagic fish species like the flounder are more hypoxia tolerant, due to the natural oxygen deprivations in their habitat [29]. In fact, changes in gene expression in response to hypoxia were generally more pronounced in the flounder (Figs. 4 and 5). These results are further supported by MANOVA statistics, which showed significant effects of species on gene expression in gills and brains (Table S4B).Previous studies have already demonstrated a pronounced hypoxia tolerance of the flounder [79], [80].

Hypoxia-adaptation in flounder is mediated by maintaining a constant O_2_ extraction rate, which is accomplished, for example, by a high concentration of blood haemoglobin as well as a high O_2_ affinity of hemoglobin [79], [81], [82]. Nevertheless, it must be taken into account that the oxygen supply to tissues depends on the cardiac performance. Comparisons of the heart rates and the stroke volumes between sluggish and active fish species resulted in 15-fold differences in the cardiac output. In general, resting cardiovascular performance of the flounder is lower compared to more active species. The stroke volume of the winter flounder (*Pleuronectes americanus*) for example is 1.5-fold lower than in Atlantic salmon (*Salmo salar*) [83]. Therefore tissues of the flounder might experience a lower oxygen supply at the same external conditions and the observed drastic changes of the expression of typical hypoxia response genes in the flounder can be interpreted as rapid defence and protection mechanisms at the cellular level [50]. This ability of an adequate response to hypoxic conditions facilitates living in coastal areas with large fluctuations in O_2_ availability.

As an invasive species in Northern America, the ruffe has already been recognised as tolerant towards a wide range of environmental stressors [84]. However, its response to hypoxia is less pronounced than in the flounder. This may be, for example, explained by the greater vertical mobility of the ruffe, which contrasts the strong demersal, and therefore putatively more hypoxic, habitat of the flounder.

### Hypoxia-response of fish is tissue-specific

We found notable differences in hypoxia response among tissues, both in terms of the pattern and the magnitude of response (Fig. 3–5). In the ruffe, the gill is by far the strongest responder (Fig. 3A) and the response in this tissue is also pronounced in the flounder (Fig. 3D). The gills are the prime site of gas exchange in fish and therefore the first target in the event of oxygen depletion [38]. In addition to their role in respiration, the gills are involved in ion exchange and acid-base regulation. These pathways are highly energy-demanding processes and therefore gills are known to be a highly oxygen-consuming tissue [85], [86]. Hence, it is reasonable that gene expression changes either secure an adequate supply of O_2_ to the mitochondria (Mb, Ngb) or fight oxidative stress (Sod2, GPx). Additionally slightly elevated mRNA levels of the apoptosis enzyme Casp3 were found in the gills of ruffe. On the one hand, this indicates a strong impact of hypoxia; on the other hand, it might be correlated with a common adaption process of carp species. An interaction of increasing apoptosis and decreasing proliferation levels, leads to a reduction in cell mass that results in an increase of the gill surface in this species [24].

Unlike in the ruffe, Casp 3 levels in the flounder suggest a stronger impact of hypoxia on the hypoxia-sensitive tissues of brains and heart. Another example is the heart of the flounder (Fig. 3F). Here, at moderate hypoxia, Mb and Ngb may enhance O_2_ supply to muscle cells and neurons, respectively. At severe hypoxia, enhanced anaerobic ATP production, as indicated by the enhanced expression of Ldha and Pkg, are required to meet the energy demands. In addition, typical stress proteins (Hsps; Sod2) are enhanced to protect the heart from the side effects of hypoxia, such as the generation of ROS.

### Hypoxia-response is stronger in the field but laboratory experiments are a good proxy

Notably, gene expression in samples from the Elbe estuary and the controlled hypoxia experiments in the laboratory showed the same patterns of expression regulations, although the laboratory setup focussing on O_2_ availability does not perfectly mirror the conditions in the Elbe environment (e.g., different temperatures). The results are supported by MANOVA, which showed a non-significant effect of the setup (field versus laboratory) on gene expression. Notable, the capture process itself seems to have no effect on gene expression levels in the ruffe or the flounder, as indicated by about the same absolute mRNA copy numbers in specimens from the Elbe and the laboratory (Fig. 2). Accordingly, the fish caught from the river most likely have similar gene expression patterns as those acclimated to laboratory conditions. However, expression changes of the hypoxia-responsive genes tended to be more pronounced in the field samples deriving from the Elbe estuary (Figs. 3 + 4). These differences in might be explained by additional extrinsic factors in the more complex natural environment, as already demonstrated in previous comparative studies [87], [88], [89].

It is well-established that besides the limited oxygen availability investigated here, other factors such as temperature and salinity affect the structure of the fish fauna [14]. For example, rising temperatures not only affect oxygen solubility itself but also cause an increase in the metabolic rate of ectotherms, which in turn leads to a higher oxygen demand [17]. Thus, hypoxia affects fish more strongly at high temperatures. One notable example in our approach is the divergent expression of Hsp27 in the ruffe, which was found much more strongly expressed in the field. This might be explained, for example, by naturally occurring temperature fluctuations that might additionally influence Hsp27 levels [90], [91].

It must also be considered that elevated temperatures in combination with high metal concentrations lead to tissue hypoxemia and aerobic energy deficiency in ectotherms [92]. Investigations on heavy metals and organic pollutants in the Elbe estuary showed highest levels between km 650.6 and 663.2, and highest predicted toxicity values between km 634 and 658 [93]. These stretches include two of the sampling stations used in this study (Pagensand NE km 662 and Wedel km 638, moderate hypoxia, see Table 1). Studies on the effects of pollutants and heavy metals on aquatic species have shown increased activities of antioxidant enzymes in the gills and livers, as well as increased occurrence of apoptotic cells in the gills [94], [95], [96], [97]. As we observed enhanced levels of Sod2 in the gills of ruffe and GPx and Casp3 in the gills of flounder from field studies, effects resulting from the interaction of elevated temperatures and pollutants cannot be excluded.

In addition, we must admit that the actual exposure times to hypoxia of specimens sampled in the field are unknown. For example, some specimens might have been preconditioned to low DO, as the first hypoxic events in the Elbe estuary usually occur in early summer. Some fish species avoid hypoxia by migrating to better-aerated regions [98], suggesting that other sampled individuals might have been exposed to hypoxia only for a short period. Such variations are beyond our experimental control. In fact, divergent life histories of individual fish in the river Elbe may at least partially explain the observed higher standard deviations of mRNA copy numbers in the field samples.

However, gene expression patterns are largely the same in samples from the field and from the laboratory. Thus, despite the additional factors that might influence gene expression changes in the field, our results suggest that laboratory experiments essentially reflect the actual patterns of hypoxia response in nature and are therefore suitable for studying the ecophysiology of fish at the molecular level.

## Supporting Information

Table S1 **Sequences of degenerated oligonucleotides.**
(DOC)

Table S2 **Sequences of qRT-PCR oligonucleotides.**
(DOC)

Table S3 **Statistical analysis (ANOVA) of qRT-PCR data.**
(DOC)

Table S4 **MANOVA of qPCR data.** A. Effects of setups (field studies versus laboratory) on gene expression in the tissues of ruffe using Pillai's trace statistics. B. Effects of species specificity on gene expression within a tissue and referred to one hypoxic condition using Pillai's trace statistics.
(DOC)
