# Supplementary material for: Molecular Response of Estuarine Fish to Hypoxia: A Comparative Study with Ruffe and Flounder from Field and Laboratory
Source: PLoS One. 2014 Mar 3;9(3):e90778. doi: 10.1371/journal.pone.0090778 (PMC3940940; doi:10.1371/journal.pone.0090778)
Supplement: Table S1 — Sequences of degenerated oligonucleotides. (DOC) [file pone.0090778.s001.doc]

**Supporting Information Table S1**. Sequences of degenerated oligonucleotides used for cloning.

| Function | Gene |  | Ruffe (5'-3' sequence) | Flounder (5'-3'sequence) |
| --- | --- | --- | --- | --- |
| Chaperone | *Hsp70* | forward | TTYCARCAYGGNAARGTNGA | TTYCARCAYGGNAARGTNGA |
|  |  | reverse | TTRTCRAARTCYTCNCCNCC | TTRTCRAARTCYTCNCCNCC |
|  | *Hsp27* | forward | ATGGCCCCACRCYCCYATGATGT | SSCARGACRRCTGGAAG |
|  |  | reverse | TACTTCTTCACCACGCCACYTTTGT | AGBKTGTATTTYCKGKTGAAG |
| Metabolic enzymes | *Pgk* | forward | ATGAGATGATCATYGGYGGYGGGMAT | ATGAGATGATCATYGGYGGYGGGMAT |
|  |  | reverse | TTTACCYTCCAGVAGCTCYAGRCTRG | TTTACCYTCCAGVAGCTCYAGRCTRG |
|  | *Ldha* | forward | acntaygtngcntggaa | ATGGACYTRCAGCATGGHAG |
|  |  | reverse | ttdatnacytcrtangc | HACRTTCACRCCACTCCA |
| Antioxidant enzymes | *SOD2* | forward | GACCTGACCTACGACTATGGT | GACCTGACCTACGACTATGGT |
|  |  | reverse | TGTACTGAAGGTARTARGCATGC | TGTACTGAAGGTARTARGCATGC |
|  | *GPx* | forward | TTYTGGGTGTKCCCTGCAATCA | TTYTGGGTGTKCCCTGCAATCA |
|  |  | reverse | CACATCCACCTTCTCGAGGAGCT | CACATCCACCTTCTCGAGGAGCT |
| Respiratory proteins | *Mb* | forward | TGGTTCTSAAGTKCTGGGGDSC | TGGTTCTSAAGTKCTGGGGDSC |
|  |  | reverse | GCAGCGTGGTYWCCYTTRGC | GCAGCGTGGTYWCCYTTRGC |
|  | *Ngb* | forward | ATCGAYGCWGCHGTSAGCCA | ATCGAYGCWGCHGTSAGCCA |
|  |  | reverse | ACAWGKTGAGCCARGCYTGRC | ACAWGKTGAGCCARGCYTGRC |
|  | *GbX* | forward | GCGAGGAGCAGATTCAGATGA | GCGAGGAGCAGATTCAGATGA |
|  |  | reverse | CTTCTTGTCGGGTCGTTCCTT | CTTCTTGTCGGGTCGTTCCTT |
| Apoptosis | *Casp 3* | forward | GGCATGAATCAGMGAAAYGGTACWG | GGCATGAATCAGMGAAAYGGTACWG |
|  |  | reverse | GTRCCTCTGCAAGCCTGRATGA | GTRCCTCTGCAAGCCTGRATGA |
| Reference genes | *β-Actin* | forward | ATCCAAATYTGYTGRAANGT | ATCCAAATYTGYTGRAANGT |
|  |  | reverse | TGGGAYGAYATGGARAARATHTGG | TGGGAYGAYATGGARAARATHTGG |
|  | *Ef1α* | forward | TCAACAAGATGGACTCCACCGAG | TCAACAAGATGGACTCCACCGAG |
|  |  | reverse | TTCAGGASACCGGTCTCAACAC | TTCAGGASACCGGTCTCAACAC |
|  | *RPLP0* | forward | AGACAGGGCCACGTGGAAG | AGACAGGGCCACGTGGAAG |
|  |  | reverse | GAGATGTTCAGCATGTTGAGCAG | GAGATGTTCAGCATGTTGAGCAG |
