# Supplementary material for: Molecular Response of Estuarine Fish to Hypoxia: A Comparative Study with Ruffe and Flounder from Field and Laboratory
Source: PLoS One. 2014 Mar 3;9(3):e90778. doi: 10.1371/journal.pone.0090778 (PMC3940940; doi:10.1371/journal.pone.0090778)
Supplement: Table S2 — Sequences of qRT-PCR oligonucleotides. (DOC) [file pone.0090778.s002.doc]

**Supporting Information Table S2A**. Sequences of oligonucleotides used for qRT-PCR (ruffe).

| Function | Gene |  | 5'-3' sequence | Accession number |
| --- | --- | --- | --- | --- |
| Transcription factor | *Hif1α* | forward | GCTTCTTCCTCCGAATGAAATGCACTC | EF100705 |
|  |  | reverse | CTGGATCATAACCCATGAGCTCAGTG |  |
| Chaperone | *Hsp70* | forward | ACAGGACCACACCCAGCTATG | HF546554 |
|  |  | reverse | TCATTGATGACATTGAATGGCCAGTGC |  |
|  | *Hsp27* | forward | CCTGGATGTCAACCACTTCTCACC | HF546553 |
|  |  | reverse | GTGCTCGTCCTTCCTTTCCTCATG |  |
| Energy metabolism | *Pgk* | forward | CTCGACCACTTTGTCCATCATGCTCT | HF546558 |
|  |  | reverse | ATGGGTTTGGACTGTGGACCAGA |  |
|  | *Ldha* | forward | TCATTGGCACGGGCACCAA | HF546555 |
|  |  | reverse | TCCGATAATCCAGCCGTGGCA |  |
| Antioxidants | *SOD2* | forward | ATGCAGAGATAATGCAGCTGCACC | HF546560 |
|  |  | reverse | GGCTACCTGTGCTGTCACATCTC |  |
|  | *GPx* | forward | CTTTGGTTCAAAGCCATTTCCAGGACG | HF546551 |
|  |  | reverse | GGACATCAGGAGAACTGCAAGAATGAAG |  |
| Respiratory proteins | *Mb* | forward | GAGGCAGACTACAACGGCAATGG | HF546556 |
|  |  | reverse | CAGCTGCATTACCAGCCAGGTC |  |
|  | *Ngb* | forward | GACCTTCACACCTTGGAGGACTT | HF546557 |
|  |  | reverse | CAGACTGCACTGCAGCATGTAGAGA |  |
|  | *GbX* | forward | AGAGACTGGAAACTCTGGCTCTGGA | HF546552 |
|  |  | reverse | AGAGACTGGAAACTCTGGCTCTGGA |  |
| Apoptosis | *Casp 3* | forward | Agatgcagccaacgcgatgaagg | HF546550 |
|  |  | reverse | TCCGTACCAAAGAACACGCCCT |  |
| Reference genes | *β-Actin* | forward | TGACGGACAGGTCATCACCATCG | HF546549 |
|  |  | reverse | TTGGCGTACAGGTCCTTACGGATG |  |
|  | *Ef1α* | forward | CACCAAGGAAGTGAGCACCTACATCAAG | HF546561 |
|  |  | reverse | CTCCTTGCGCTCAACCTTCCATC |  |
|  | *RPLP0* | forward | GACAATGTGGGCTCCAAGCAGATG | HF546559 |
|  |  | reverse | ATCTCAGCCAGATCCTCCTTGGT |  |

**Supporting Information Table S2B**. Sequences of oligonucleotides used for qRT-PCR (flounder).

| Function | Gene |  | 5'-3' sequence | Accession number |
| --- | --- | --- | --- | --- |
| Transcription factor | *Hif1α* | forward | GCTTCTTCCTCCGAATGAAATGCACTC | EF100705 |
|  |  | reverse | CTGGATCATAACCCATGAGCTCAGTG |  |
| Chaperone | *Hsp70* | forward | ACAGGACCACACCCAGCTATG | HF546568 |
|  |  | reverse | TCATTGATGACATTGAATGGCCAGTGC |  |
| Energy metabolism | *Pgk* | forward | CTCGACCACTTTGTCCATCATGCTCT | HF546572 |
|  |  | reverse | ATGGGTTTGGACTGTGGACCAGA |  |
|  | *Ldha* | forward | GACTACAGTGTGACAGCCAACTCCA | HF546569 |
|  |  | reverse | GTTGGAAACCACCATCAGGATGCAG |  |
| Antioxidants | *SOD2* | forward | ACGGAGGAGAAGTATCACGAGGCA | HF546574 |
|  |  | reverse | GAGACAGGTTTGTCCAGAAGATGGTGTG |  |
|  | *GPx* | forward | CTTTGGTTCAAAGCCATTTCCAGGACG | HF546566 |
|  |  | reverse | GGACATCAGGAGAACTGCAAGAATGAAG |  |
| Respiratory proteins | *Mb* | forward | CACTCAGAAGCTGTTCCCCAACTTTG | HF546570 |
|  |  | reverse | CCTTCAGCAGGTCACCAAGTTTCTTC |  |
|  | *Ngb* | forward | GACCTTCACACCTTGGAGGACTT | HF546571 |
|  |  | reverse | CAGACTGCACTGCAGCATGTAGAGA |  |
|  | *GbX* | forward | AGAGACTGGAAACTCTGGCTCTGGA | HF546565 |
|  |  | reverse | GCTGGTCACATACTGGAACATGGTCTTC |  |
| Apoptosis | *Casp 3* | forward | CGGCCAACGCGATGAAAGTCTT | HF546563 |
|  |  | reverse | CTGTGATCCTCCTTTGATACGCCAAG |  |
| Reference genes | *β-Actin* | forward | TGACGGACAGGTCATCACCATCG | HF546562 |
|  |  | reverse | TTGGCGTACAGGTCCTTACGGATG |  |
|  | *Ef1α* | forward | CACCAAGGAAGTGAGCACCTACATCAAG | HF546564 |
|  |  | reverse | CTCCTTGCGCTCAACCTTCCATC |  |
|  | *RPLP0* | forward | CTTCATCGTGGGGGCAGACAA | HF546573 |
|  |  | reverse | ACGGATGGCTTTACGCATCATGGT |  |
