# Supplementary material for: Molecular Response of Estuarine Fish to Hypoxia: A Comparative Study with Ruffe and Flounder from Field and Laboratory
Source: PLoS One. 2014 Mar 3;9(3):e90778. doi: 10.1371/journal.pone.0090778 (PMC3940940; doi:10.1371/journal.pone.0090778)
Supplement: Table S3 — Statistical analysis (ANOVA) of qRT-PCR data. (DOC) [file pone.0090778.s003.doc]

**Supporting Information Table S3A.** Statistical analysis (ANOVA) of qRT-PCR data in gills.

| **Gills** | | | **Elbe** | | | | **Laboratory** | | | | | |
| --- | --- | --- | --- | --- | --- | --- | --- | --- | --- | --- | --- | --- |
| **Function** | **Gene** |  | **Mild hypoxia** | | **Moderate hypoxia** | | **Mild hypoxia** | | **Moderate hypoxia** | | **Severe hypoxia** | |
|  |  |  | **Ruffe** | **Flounder** | **Ruffe** | **Flounder** | **Ruffe** | **Flounder** | **Ruffe** | **Flounder** | **Ruffe** | **Flounder** |
| **Transcription factor** | ***Hif1a*** | **RE ±SE** | 1.85±0.55 | 0.24 ±0.06 | 2.5±1.16 | 1.71 ±1.06 | 0.99 | - | 1.78 | - | 2.05 ±0.66 | 1.67 ±0.33 |
|  |  | **Significance** | 0.877 | 0.017 | 0.348 | 0.899 |  | - | 0.618 | - | 0.701 | 0.168 |
| **Chaperone** | ***Hsp70*** | **RE ±SE** | 1.15 ±0.55 | 0.35 ±0.17 | 0.43 ±0.11 | 2.84 ±1.95 | 0.8 ±0.09 | - | 1.22 ±0.21 | - | 2.22 ±0.56 | 1.34 ±0.31 |
|  |  | **Significance** | 0.991 | 0.149 | 0.017 | 0.793 | 0.111 | - | 0.872 | - | 0.43 | 0.634 |
|  | ***Hsp27*** | **RE ±SE** | 1.06 ±0.51 | - | 50.1 ±3.8 | - | 2.23 ±0.32 | - | 2.45 ±0.33 | - | 4.56 ±1.7 | - |
|  |  | **Significance** | 0.999 | - | 0.001 | - | 0.041 | - | 0.024 | - | 0.297 | - |
| **Respiratory protein** | ***Mb*** | **RE ±SE** | 7.16 ±4.13 | 118 ±52.5 | 10.5 ±7.0 | 160 ±61.7 | 0.55 ±0.13 | - | 3.3 ±0.65 | - | 5.7 ±1.3 | 3.78 ±1.71 |
|  |  | **Significance** | 0.566 | 0.351 | 0.722 | 0.284 | 0.069 | - | 0.076 | - | 0.064 | 0.518 |
|  | ***Ngb*** | **RE ±SE** | 37.5 ±36 | 12.1 ±7.22 | 3.7 ±1.3 | 41.4 ±18.9 | 0.55 ±0.15 | - | 7.27 ±2.5 | - | 2.27 ±0.83 | 2.14 ±0.51 |
|  |  | **Significance** | 0.344 | 0.553 | 0.773 | 0.372 | 0.121 | - | 0.208 | - | 0.506 | 0.488 |
|  | ***GbX*** | **RE ±SE** | 35.6 ±33.8 | 2.28 ±0.98 | 2.61 ±0.59 | 1.38 ±0.57 | 1.36 ±0.36 | - | 1.44 ±0.4 | - | 2.2 ±0.49 | 0.96 ±0.51 |
|  |  | **Significance** | 0.45 | 0.638 | 0.682 | 0.901 | 0.766 | - | 0.706 | - | 0.216 | 0.999 |
| **Energy metabolism** | ***Ldha*** | **RE ±SE** | 2.03 ±1.6 | 1.79 ±0.04 | 1.97 ±0.38 | 1.66 ±0.51 | 0.93 ±0.09 | - | 2.15 ±0.56 | - | 3.51 ±1.14 | 1.67 ±0.44 |
|  |  | **Significance** | 0.908 | 0.7 | 0.117 | 0.641 | 0.887 | - | 0.302 | - | 0.267 | 0.058 |
|  | ***Pgk*** | **RE ±SE** | 4.51 ±3.5 | 0.37 ±0.1 | 2.21 ±0.25 | 0.53 ±0.07 | 1.03 ±0.09 | - | 1.59 ±0.25 | - | 0.4 ±0.05 | 1.45 ±0.08 |
|  |  | **Significance** | 0.772 | 0.061 | 0.046 | 0.054 | 0.994 | - | 0.223 | - | 0.0001 | 0.076 |
| **Antioxidant** | ***Sod2*** | **RE ±SE** | 3.46 ±2.8 | 0.62 ± 0.1 | 1.83 ±0.53 | 0.66 ±0.06 | 1.68 ±0.41 | - | 1.67 ±0.24 | - | 2.48 ±0.45 | 1.22 ±0.55 |
|  |  | **Significance** | 0.818 | 0.142 | 0.208 | 0.073 | 0.444 | - | 0.163 | - | 0.095 | 0.752 |
|  | ***GPx*** | **RE ±SE** | 0.77 ±0.31 | 1.22 ±0.31 | 1.52 ±0.19 | 6.34 ±4.46 | 0.64 ±0.12 | - | 0.68 ±0.11 | - | 2.58 ±0.75 | 1.64 ±0.63 |
|  |  | **Significance** | 0.87 | 0.887 | 0.2 | 0.684 | 0.137 | - | 0.142 | - | 0.291 | 0.759 |
| **Apoptosis** | ***Casp 3*** | **RE ±SE** | 1.04 ±0.62 | 1.77 ±0.4 | 1.93 ±0.41 | 2.73 ±0.30 | 1.96 ±0.53 | - | 1.37 ±0.23 | - | 2.9 ±0.77 | 1.1 ±0.07 |
|  |  | **Significance** | 0.999 | 0.471 | 0.286 | 0.200 | 0.383 | - | 0.473 | - | 0.208 | 0.55 |

**Supporting Information Table S3B.** Statistical analysis (ANOVA) of qRT-PCR data in brain.

| **Brain** | | | **Elbe** | | | | **Laboratory** | | | | | |
| --- | --- | --- | --- | --- | --- | --- | --- | --- | --- | --- | --- | --- |
| **Function** | **Gene** |  | **Mild hypoxia** | | **Moderate hypoxia** | | **Mild hypoxia** | | **Moderate hypoxia** | | **Severe hypoxia** | |
|  |  |  | **Ruffe** | **Flounder** | **Ruffe** | **Flounder** | **Ruffe** | **Flounder** | **Ruffe** | **Flounder** | **Ruffe** | **Flounder** |
| **Transcription factor** | ***Hif1a*** | **RE ±SE** | 0.89 ±0.15 | 2.33 ±0.65 | 0.51 ±0.16 | 0.5 ±0.04 | 1.06 ±0.24 | - | 0.96 ±0.19 | - | 1.52±0.36 | 1.92 ±0.33 |
|  |  | **Significance** |  | 0.319 |  | 0.019 | 0.994 |  | 0.845 |  | 0.847 | 0.191 |
| **Chaperone** | ***Hsp70*** | **RE ±SE** | 0.95 ±0.06 | 1.72 ±0.85 | 0.51 ±0.18 | 0.85 ±0.17 | 0.68 ±0.17 | - | 0.71 ±0.09 | - | 1.9 ±0.44 | 2.1 ±0.31 |
|  |  | **Significance** | 0.824 | 0.812 | 0.109 | 0.831 | 0.982 | - | 0.693 | - | 0.485 | 0.167 |
|  | ***Hsp27*** | **RE ±SE** | 1.0 ±0.09 | - | 0.61 ±0.27 | - | 1.69 ±0.36 | - | 1.99 ±0.41 | - | 1.71 ±0.15 | - |
|  |  | **Significance** | 0.999 | - | 0.057 | - | 0.279 | - | 0.163 | - | 0.009 | - |
| **Respiratory protein** | ***Mb*** | **RE ±SE** | 0.51 ±0.2 | 41.6 ±27.4 | 1.01 ±0.34 | 222.3 ±137 | 173 ±24.0 | - | 0.9 ±0.32 | - | 1.4 ±0.16 | 9.8 ±8.7 |
|  |  | **Significance** | 0.314 | 0.569 | 0.999 | 0.522 | 0.009 | - | 0.987 | - | 0.344 | 0.76 |
|  | ***Ngb*** | **RE ±SE** | 2.3 ±0.89 | 10.1 ±2.24 | 2.63 ±1.1 | 3.48 ±1.05 | 1.36 ±0.25 | - | 0.95 ±0.16 | - | 2.84 ±0.54 | 2.24 ±0.51 |
|  |  | **Significance** | 0.405 | 0.254 | 0.677 | 0.325 | 0.555 | - | 0.987 | - | 0.085 | 0.249 |
|  | ***GbX*** | **RE ±SE** | 1.18 ±0.74 | 55.6 ±0.16 | 2.7 ±1.04 | 8.88 ±5.06 | 1.1 ±0.13 | - | 0.13 ±0.08 | - | 1.63 ±0.5 | 2.5 ±0.42 |
|  |  | **Significance** | 0.993 | 0.011 | 0.476 | 0.846 | 0.956 | - | 0.002 | - | 0.224 | 0.109 |
| **Energy metabolism** | ***Ldha*** | **RE ±SE** | 0.35 ±0.12 | 0.61 ±0.15 | 0.31 ±0.09 | 1.21 ±0.67 | 1.1 ±0.23 | - | 0.89 ±0.1 | - | 2.83 ±0.65 | 2.46 ±0.44 |
|  |  | **Significance** | 0.079 | 0.269 | 0.013 | 0.987 | 0.957 | - | 0.724 | - | 0.146 | 0.128 |
|  | ***Pgk*** | **RE ±SE** | 0.74 ±0.25 | 1.05 ±0.34 | 0.53 ±0.22 | 0.84 ±0.28 | 1.23 ±0.2 | - | 1.17 ±0.21 | - | 0.93 ±0.12 | 1.1±0.08 |
|  |  | **Significance** | 0.764 | 0.998 | 0.291 | 0.937 | 0.666 | - | 0.863 | - | 0.939 | 0.772 |
| **Antioxidant** | ***Sod2*** | **RE ±SE** | 0.64 ±0.22 | 1.81 ±0.69 | 0.62 ±0.16 | 1.13 ±0.12 | 1.4 ±0.27 | - | 1.0 ±0.14 | - | 1.82 ±0.36 | 1.86 ±0.55 |
|  |  | **Significance** | 0.514 | 0.694 | 0.226 | 0.743 | 0.512 | - | 0.999 | - | 0.242 | 0.547 |
|  | ***GPx*** | **RE ±SE** | 0.74 ±0.31 | 2.05 ±0.96 | 0.62 ±0.19 | 0.97 ±0.29 | 0.83 ±0,09 | - | 1.9 ±0.44 | - | 1.74 ±0.49 | 1.03 ±0.39 |
|  |  | **Significance** | 0.239 | 0.731 | 0.357 | 0.999 | 0.417 | - | 0.309 | - | 0.522 | 0.999 |
| **Apoptosis** | ***Casp 3*** | **RE ±SE** | 0.64 ±0.62 | 8.57 ±3.01 | 0.56 ±1.1 | 3.92 ±0.36 | 5.52 ±1.51 | - | 1.23 ±0.17 | - | 2.46 ±0.52 | 2.06 ±0.24 |
|  |  | **Significance** | 0.223 | 0.296 | 0.256 | 0.038 | 0.126 | - | 0.588 | - | 0.143 | 0.064 |

**Supporting Information Table S3C. Statistical analysis (ANOVA) of qRT-PCR data in heart.**

| **Heart** | | | **Elbe** | | | | **Laboratory** | | | | | |
| --- | --- | --- | --- | --- | --- | --- | --- | --- | --- | --- | --- | --- |
| **Function** | **Gene** |  | **Mild hypoxia** | | **Moderate hypoxia** | | **Mild hypoxia** | | **Moderate hypoxia** | | **Severe hypoxia** | |
|  |  |  | **Ruffe** | **Flounder** | **Ruffe** | **Flounder** | **Ruffe** | **Flounder** | **Ruffe** | **Flounder** | **Ruffe** | **Flounder** |
| **Transcription factor** | ***Hif1a*** | **RE ±SE** | 0.76±0.09 | 0.3 ±0.16 | 0.78±0.29 | 0.53 ±0.11 | 0.59±0.11 | - | 0.95±0.22 | - | 1.14±0.34 | 1.95 ±0.33 |
|  |  | **Significance** | 0.42 | 0.256 | 0.999 | 0.258 | 0.999 | - | 0.793 | - | 0.072 | 0.523 |
| **Chaperone** | ***Hsp70*** | **RE ±SE** | 1.18 ±0.01 | 0.18 ±0.15 | 0.6 ±0.16 | 0.76 ±0.01 | 0.44 ±0.06 | - | 0.61 ±0.09 | - | 1.79 ±0.51 | 2.34 ±0.84 |
|  |  | **Significance** | 0.061 | 0.209 | 0.135 | 0.043 | 0.011 | - | 0.054 | - | 0.83 | 0.604 |
|  | ***Hsp27*** | **RE ±SE** | 0.03 ±0.01 | - | 3.61 ±0.36 | - | 0.65 ±0.08 | - | 0.92 ±0.16 | - | 1.55 ±0.34 | - |
|  |  | **Significance** | 0.009 | - | 0.007 | - | 0.075 | - | 0.947 | - | 0.475 | - |
| **Respiratory protein** | ***Mb*** | **RE ±SE** | 0.001 ±0.0001 | 1.45 ±0.59 | 1,47 ±0.43 | 0.51 ±0.05 | 0.56 ±0.07 | - | 0.76 ±0.09 | - | 0.8 ±0.23 | 1.23 ±0.06 |
|  |  | **Significance** | 0.001 | 0.866 | 0.705 | 0.111 | 0.011 | - | 0.212 | - | 0.817 | 0.311 |
|  | ***Ngb*** | **RE ±SE** | 7.32 ±5.4 | 1.94 ±0.13 | 12.6 ±4.8 | 4.78 ±2.77 | 0.53 ±0.08 | - | 0.96 ±0.08 | - | 0.85 ±0.11 | 2.82 ±0.73 |
|  |  | **Significance** | 0.316 | 0.156 | 0.682 | 0.672 | 0.032 | - | 0.99 | - | 0.696 | 0.43 |
|  | ***GbX*** | **RE ±SE** | 0.46 ±0.19 | 4.53 ±2.25 | ±1,08 | 2.86 ±2.58 | 1.11 ±0.26 | - | 3.96 ±1.38 | - | 0.95 ±0,25 | 6.52 ±0.71 |
|  |  | **Significance** | 0.398 | 0.611 | 0.597 | 0.883 | 0.983 | - | 0.316 | - | 0.795 | 0.148 |
| **Energy metabolism** | ***Ldha*** | **RE ±SE** | 0.1 ±0.02 | 0.11 ±0.04 | 0.67 ±0.22 | 0.55 ±0.31 | 0.73 ±0.12 | - | 1.47 ±0.34 | - | 2.63 ±0.87 | 7.05 ±0.16 |
|  |  | **Significance** | 0.026 | 0.048 | 0.55 | 0.642 | 0.269 | - | 0.572 | - | 0.397 | 0.03 |
|  | ***Pgk*** | **RE ±SE** | 0.47 ±0.0007 | 0.54 ±0.1 | 1.28 ±0.26 | 0.83 ±0.07 | 0.84 ±0.09 | - | 0.97 ±0.31 | - | 0.2 ±0.06 | 2.53 ±0.79 |
|  |  | **Significance** | 0.002 | 0.485 | 0.724 | 0.454 | 0.365 | - | 0.999 | - | 0.002 | 0.536 |
| **Antioxidant** | ***Sod2*** | **RE ±SE** | 0.46 ±0.02 | 0.33 ±0.16 | 0.95 ±0.16 | 0.54 ±0.09 | 0.77 ±0.15 | - | 0.88 ±0.08 | - | 1,27 ±0.46 | 1.8 ±0.14 |
|  |  | **Significance** | 0.051 | 0.266 | 0.991 | 0.235 | 0.53 | - | 0.52 | - | 0.923 | 0.205 |
|  | ***GPx*** | **RE ±SE** | 1.24 ±0.06 | 0.89 ±0.21 | 1.82 ±0.85 | 1.23 ±0.51 | 0.83 ±0.13 | - | 2.0 ±0.65 | - | 0.99 ±0.28 | 0.43 ±0.09 |
|  |  | **Significance** | 0.296 | 0.946 | 0.773 | 0.959 | 0.586 | - | 0.51 | - | 0.999 | 0.2 |
| **Apoptosis** | ***Casp 3*** | **RE ±SE** | 1.1 ±0.11 | 4.9 ±2.94 | 0.61 ±0.05 | 5.28 ±0.26 | 7.15 ±1.52 | - | 0.87 ±0.07 | - | 1.99 ±0.67 | 4.36 ±0.44 |
|  |  | **Significance** | 0.927 | 0.679 | 0.45 | 0.07 | 0.079 | - | 0.357 | - | 0,541 | 0.064 |
