# Supplementary material for: Molecular Response of Estuarine Fish to Hypoxia: A Comparative Study with Ruffe and Flounder from Field and Laboratory
Source: PLoS One. 2014 Mar 3;9(3):e90778. doi: 10.1371/journal.pone.0090778 (PMC3940940; doi:10.1371/journal.pone.0090778)
Supplement: Table S4 — MANOVA of qPCR data. A. Effects of setups (field studies versus laboratory) on gene expression in the tissues of ruffe using Pillai's trace statistics. B. Effects of species specificity on gene expression within a tissue and referred to one hypoxic condition using Pillai's trace statistics. (DOC) [file pone.0090778.s004.doc]

**Table S4. MANOVA of qPCR data. A.** Effects of setups (field studies versus laboratory) on gene expression in the tissues of ruffe using Pillai's trace statistics.

|  | Effect | F-value | P-value |
| --- | --- | --- | --- |
| Gills | Mild hypoxia | 22.4 (4,1) | 0.157 |
|  | Moderate hypoxia | 0.62 (3,1) | 0,711 |
| Brain | Mild hypoxia | 4.4 (5,1) | 0.354 |
|  | Moderate hypoxia | 164.6 (3,1) | 0.06 |
| Heart | Mild hypoxia | 53.0 (3,1) | 0.101 |
|  | Moderate hypoxia | 27.8 (3,1) | 0.133 |

**Table S4. MANOVA of qPCR data. B.** Effects of species specificity on gene expression within a tissue and referred to one hypoxic condition using Pillai's trace statistics.

|  | Effect | F-value | P-value |
| --- | --- | --- | --- |
| Gills | Mild hypoxia field | 0.55 (3,1) | 0.731 |
|  | Moderate hypoxia field | 30.1 (3,2) | **0.032** |
|  | Severe hypoxia lab | 154.7 (3,1) | 0.427 |
| Brain | Mild hypoxia field | 17.7 (4,1) | 0.225 |
|  | Moderate hypoxia field | 414.0 (4,1) | **0.037** |
|  | Severe hypoxia lab | 317.4 (4,1) | **0.042** |
| Heart | Mild hypoxia field | 0.5 (3,1) | 0.77 |
|  | Moderate hypoxia field | 1.34 (3,1) | 0.52 |
|  | Severe hypoxia lab | 1.06 (3,1) | 0.59 |
